# The association of a genetic variant in CDKN2A/B gene and the risk of colorectal cancer

**DOI:** 10.17179/excli2020-2051

**Published:** 2020-09-14

**Authors:** Farzad Rahmani, Amir Avan, Forouzan Amerizadeh, Gordon A. Ferns, Sahar Talebian, Soodabeh Shahidsales

**Affiliations:** 1 Iranshahr University of Medical Sciences, Iranshahr, Iran; 2 Cancer Research Center, Mashhad University of Medical Sciences, Mashhad, Iran; 3 Metabolic Syndrome Research Center, Mashhad University of Medical Sciences, Mashhad, Iran; 4 Brighton & Sussex Medical School, Division of Medical Education, Falmer, Brighton, Sussex BN1 9PH, UK

**Keywords:** colorectal cancer, CDKN2A/B, polymorphism

## Abstract

Colorectal cancer is among the most aggressive tumors, and its development involves an interplay between various genetic and environmental familial risk factors. Several genetic polymorphisms have been reported to be associated with colorectal cancer in recent studies. In this current study, we aimed to evaluate the possible relationship between a CDKN2A/B, single nucleotide polymorphisms (SNP) (rs10811661), with the risk of colorectal cancer. A total of 541 individuals with, or without cancer were recruited. DNA was extracted, and genotyped using a Taq-Man based real‐time PCR method. The rs10811661 SNP was associated with an increased risk of colorectal cancer (additive model: OR=3.46, CI= 1.79-6.69, p<0.0001 and recessive model: 5.72, CI= 3.12-10.49, p<0.0001). The distribution of minor alleles in the total population for homozygote allele was 9.2 %, while this was 20.1 % for heterozygotes. In summary, our findings indicate that the rs10811661 polymorphism of the CDKN2A/B gene was strongly related to the occurrence of colorectal cancer suggesting its potential role as a prognostic biomarker for the management of colorectal cancer.

## Introduction

Colorectal cancer (CRC) is among the most common tumors worldwide with over 700,000 deaths annually (Soleimani et al., 2019[30]; Gabelloni et al., 2019[7]). Attempts have been made to identify new markers for colorectal cancer; few of them are approved for cancer diagnosis and prognosis. Recently, multiple molecular mechanisms have been reported in CRC tumorigenesis including inactivation of tumor suppressor genes (Rahmani et al., 2018[20]; Soleimani et al., 2018[28], 2020[29]; Bahreyni et al., 2018[2]; Binabaj et al., 2018[3]). Several genetic association studies have suggested that genetic variations on chromosome 9p21 may be involved in various malignancies including leukemia, glioma, ovarian, breast and pancreatic cancers (Congrains et al.,2013[5]; Dębniak et al., 2005[6]; Sherborne et al., 2010[27]; Qiu et al., 2015[19]; Gu et al., 2013[9]; Seifi et al., 2019[24]; Abdeahad et al., 2020[1]). This region encodes for cyclin‐dependent kinase inhibitors A and B known as CDKN2A/B which contributes in various metabolic and pathological disorders such as diabetes, metabolic syndrome, cardiovascular and Alzheimer diseases (Hannou et al., 2015[10]; Mehramiz et al., 2018[16]; Yu et al., 2010[34]; Zeggini et al., 2007[35]). Recent data has shown that the CDKN2A/B gene can regulate cell growth by arresting the cell cycle at G1 phase. The cell cycle progression at the G1 phase is mainly modulated by p14^ARF^, p15^INK4B^ and 16^INK4A^ proteins (Hannou et al., 2015[10]; Nielsen et al., 2001[17]; McLendon et al., 2008[15]). The p15^INK4B^ and 16^INK4A ^tumor suppressor proteins induce cell cycle arrest by downregulating cyclin-dependent kinase 4 and 6 (CDK4, 6) while the p14^ARF^ protein promotes apoptosis and cell cycle arrest by promoting mdm2 -p53 signaling pathway (Krimpenfort et al., 2019[11]; Sharpless and DePinho, 1999[26]). It has been shown that while the tumor suppressors p14^ARF^ and p16^INK4A ^are encoded by CDKN2A, the p15^INK4B^ protein is encoded by CDKN2B (Sharpless and DePinho, 1999[26]).

There is emerging evidence that genes at the CDKN2A/B locus genes are mutated or deleted in several human malignancies. There are various SNPs in the CDKN2A/2B locus resulting in downregulation of their expression and inducing tumor cell proliferation and progression (Zeggini et al., 2007[35]; Royds et al., 2016[22]). Recent data have shown that the CDKN2A/B deletion was correlated with poor prognosis and lower survival in patients with cutaneous T-cell lymphomas (Laharanne et al., 2010[12]). Another large-scale meta-analysis study performed by Lu et al. on the relationship between CDKN2A/B gene polymorphism rs4977756 and the risk of glioma was assessed in 18893 individuals with, or without cancer. This analysis showed that the rs4977756 polymorphism was significantly associated with the risk of glioma (Lu et al., 2015[14]). Consistent with these studies, the correlation of CDKN2A/B gene (rs10811661) polymorphism was investigated in 564 breast cancer patients and results revealed that individuals with the TT genotype had greater susceptibility to breast cancer (ShahidSales et al., 2018[25]). The association of two SNP of the CDKN2A/B locus (rs1333049 and rs10811661) and clinical manifestations of esophageal squamous cell carcinoma (ESCC) was assessed and suggested that the CC genotype of rs1333049 polymorphism was related to a poorer prognosis and lower overall survival in patient with ESCC (Ghobadi et al., 2019[8]).

Thus, we aimed to explore the association of CDKN2A/B gene (rs10811661) polymorphism in Iranian colorectal cancer patients.

## Material and Methods

### Patient samples

A total of 541 individuals (132 colorectal cancer patients and 409 matched controls) were recruited from Omid or Ghaem Hospitals of Mashhad University of Medical Sciences (MUMS). The cases with colorectal cancer were diagnosed with colonoscopy findings followed by histopathological analysis (between 2016 to 2018). All patients provided written, informed consent, and the study was approved by the Ethics Committee of MUMS.

### DNA genotyping

DNA genotyping was performed on genomic DNAs obtained from whole blood leukocytes. First, DNA samples were extracted by commercial Extraction Kit (Parstous, Mashhad, Iran) according to manufacturer's instruction. Next, the quality and quantity of extracted DNA were studied by spectrophotometry (NanoDrop-Thermo Scientific, USA). Genotyping of CDKN2A/B variants were performed by qPCR method and the PCR mixture consisted of 20 ng DNA+ 2.13 µl TaqMan® Master Mix with specific probes in 12 µl total volume (Rahmani et al., 2020[21]). The ABI PRISM- 7500 instrument was used to determine the sample genotype.

### Statistics

Kolmogorov-Smirnov tests were used to assess the normality of the distribution of data within the subgroups. The normally distributed continuous data were tested by Student's t-tests. The frequencies of CDKN2A/B rs10811661 polymorphism were compared using Pearson χ2 tests and the Hardy-Weinberg test was evaluated through comparing the genotype frequencies via Pearson χ2 test. The demographic and clinicopathological data of 132 cases were evaluated in various genotypes using independent t-test and Pearson chi square tests. The association between the CC and CT genotypes, related to the risk genotype TT on additive and recessive models were evaluated by logistic regression. Odds ratios and 95 % confidence interval for each genotype on rs10811661 was assessed by multivariate logistic regression models. The data analysis was conducted by SPSS- 22 software. p-values less than 0.05 were taken as statistically significant and all tests were two-sided.

## Results

### Association of the CDKN2A/B (rs10811661) with clinical characteristics

Demographic and clinicopathological data including age, weight, metastasis, and tumor grade were investigated in colorectal cancer patients (Table 1(Tab. 1)). No relationship was found for TT and TC/CC genotypes with age, weight, metastasis, and tumor grade in recessive genetic model (p > .05) (Table 2(Tab. 2)).

### Association of the CDKN2A/B (rs10811661) with the risk of colorectal cancer

In order to investigate the correlation between CDKN2A/B polymorphism, rs10811661, and susceptibility to colorectal cancer, genotyping was performed on genomic DNAs obtained from whole blood leukocytes. Also, the Hardy-Weinberg equilibrium was assessed in the population (Table 3(Tab. 3)). The distribution of CDKN2A/B genotypes in healthy and CRC samples is presented in Table 3(Tab. 3). In the total population, the frequencies of TT, CT, CC genotype calculated 9.2, 20.1, and 70.6 %. This genotype distribution was in accordance with the H-W equilibrium. Our results showed that subjects with TT genotype of CDKN2A/B rs10811661 have an increased risk of CRC (p<0.0001) in comparison with the healthy controls (Table 3(Tab. 3)). Additionally, the logistic regression analysis on recessive and additive models indicate that individuals with the CC/CT genotypes had a lower susceptibility for colorectal cancer (recessive model: OR=5.72, CI= 3.12-10.49, p<0.0001 and additive model: OR=3.46, CI= 1.79-6.69, p<0.0001) compared to TT carriers. In addition, no significant correlation was found in dominant genetic model (Table 4(Tab. 4)). 

## Discussion

In conclusion, our results suggest a relationship between a polymorphism at the CDKN2A/B gene (rs10811661) locus and a poor prognosis in patients with colorectal cancer. Individuals with a TT genotype had a greater susceptibility for colorectal cancer. In line with our results, recent studies have also indicated the prognostic role of CDKN2A/B in pancreatic, lung, breast, melanoma and ovarian cancers (Qiu et al., 2015[19]; Seifi et al., 2019[24]; Campa et al., 2016[4]; Schuster et al., 2014[23]). This observation may be explained by the role of CDKN2A/B in suppressing cellular proliferation and inducing tumor cell death. There are several studies demonstrated that methylation or ANRIL regulation may downregulate CDKN2A/B and its downstream tumor suppressors (p14^ARF^ and p16^INK4A^), resulting in tumor formation and progression. ANRIL has been shown to have a major role in promoting transcriptional repressors involved in downregulation of the CDKN2A/B genes resulting in genetic susceptibility to various cancers (Congrains et al., 2013[5]; Yap et al., 2010[33]; Popov and Gil, 2010[18]). In agreement with these data, Sun et al. examined the expression of ANRIL in 97 paired tumoral and non-tumoral CRC tissue samples. They found that the over-expression of ANRIL in tumor tissues was related to lower survival in CRC patients. Moreover their *in vitro* results demonstrated that downregulation of ANRIL in CRC cell lines decreased cellular proliferation and invasion (Sun et al., 2016[31]). In another study, the correlation between ANRIL expression and clinicopathological features of CRC was assessed in 108 patients. Their results demonstrated that the over-expression of ANRIL in CRC patient may be considered as a risk factor for poor prognosis and tumor metastasis (Sun et al., 2016[32]). However, the potential role of ANRIL in colorectal tumorigenesis still requires to be determined. Recently, a large-scale genome wide association study was performed to explore the correlation of 9p21 locus SNPs and the risk of neoplastic transformation in multiple cancers. Their data revealed that there are various genetic variations in this region related to the development of several types of cancers (Li et al., 2014[13]). In line with this, Gu et al. analyzed 203 SNPs on the 9p21.3 region in several cancers including colorectal cancer. Their findings indicated that the genetic variants in CDKN2A may be related to the risk of colorectal cancer and other tumors (Gu et al., 2013[9]).

In agreement with these observations, our data support a significant correlation between the CDKN2A/B gene polymorphism, rs10811661, and colorectal cancer. Further studies in a larger sample are required to validate our results and investigate the prognostic potential of rs10811661 in determining the risk of developing colorectal cancer.

## Notes

Farzad Rahmani and Amir Avan contributed equally as first author.

## Funding

This study was supported by grant from Mashhad University of Medical Sciences.

## Conflict of interest

The authors have no conflicts of interest to declare.

## Figures and Tables

**Table 1:**
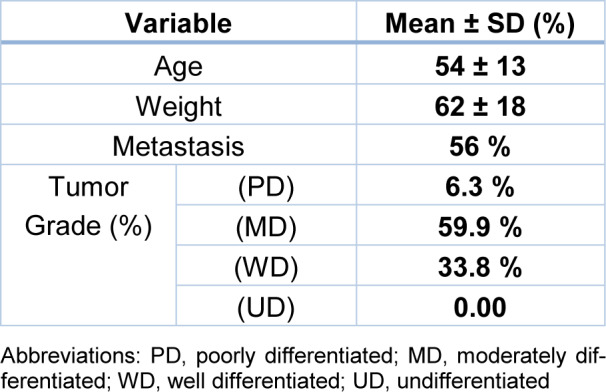
Clinicopathological features of patients with colorectal cancer (n=132)

**Table 2:**
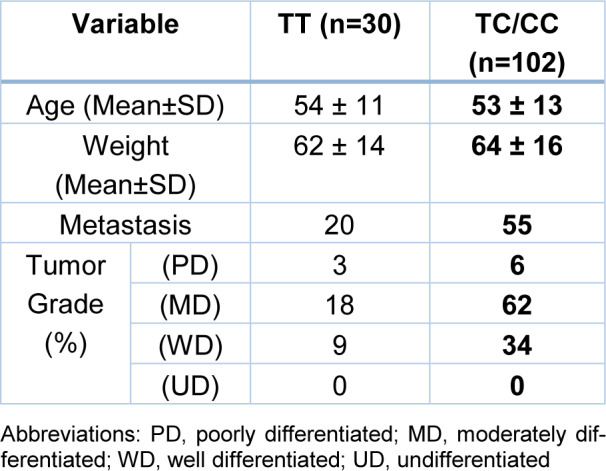
Baseline and clinicopathological characteristics of patients with colorectal cancer under the recessive model

**Table 3:**
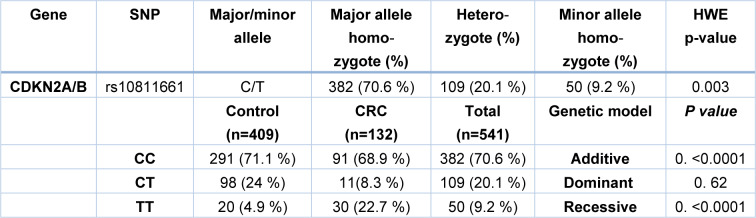
Allele and genotype frequencies of CDKN2A/B rs10811661 polymorphism

**Table 4:**
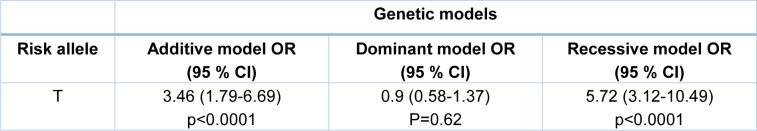
Multivariable logistic regression analysis of rs10811661 polymorphism and colorectal cancer under different genetic models
